# Multiple roles of MicroRNAs in melanoma: biomarkers for diagnosis, prognosis, and treatment prediction

**DOI:** 10.3389/fimmu.2026.1826584

**Published:** 2026-04-29

**Authors:** Zicheng Huang, Yuanke He, Qianyu Chen, Ting Hu

**Affiliations:** 1 The First Clinical College, Chongqing Medical University, Chongqing, China; 2 State Key Laboratory of Molecular Oncology, National Cancer Center/National Clinical Research Center for Cancer/Cancer Hospital, Chinese Academy of Medical Sciences and Peking Union Medical College, Beijing, China

**Keywords:** diagnostic biomarker, melanoma, microRNA, prognostic assessment, treatment prediction, tumor immune microenvironment

## Abstract

Melanoma, a highly malignant form of skin cancer, continues to rise in incidence worldwide. Early diagnosis and accurate prognostic assessment are critical to reducing melanoma-related mortality rates. MicroRNAs (miRNAs), a class of non-coding RNAs approximately 22 nucleotides in length, play pivotal roles in melanoma initiation, progression, invasion, and metastasis by regulating the expression of target genes. Beyond their cell-intrinsic functions, miRNAs are increasingly recognized as critical regulators of the tumor immune microenvironment (TIME). They modulate anti-tumor immune responses by influencing immune checkpoint expression, immune cell recruitment and function, and intercellular communication via extracellular vesicles (EVs). This comprehensive review explores the multifaceted functions of miRNAs in melanoma, highlighting their potential as diagnostic markers, prognostic indicators, and predictors of therapeutic response. It also addresses current research challenges and explores future directions, including the integration of AI-powered spatial transcriptomics to decipher the complex, context-dependent networks of miRNAs within the TIME, offering a theoretical foundation and novel insights for precision medicine in melanoma treatment.

## 1 Introduction

Melanoma, originating from neural crest-derived melanocytes, represents one of the most malignant subtypes of skin cancer. The global incidence rate is increasing by 3%-5% annually, posing a substantial threat to public health (1). Patients diagnosed with early-stage melanoma (Stages I-II) have a 5-year survival rate exceeding 90% following surgical resection. However, once the disease advances to Stages III or IV with lymph node or distant organ metastasis, the 5-year survival rate drops below 30%. Moreover, recurrence and drug resistance frequently occur after treatment, further worsening survival outcomes (2). Early diagnosis is therefore critical for improving melanoma prognosis. Currently, clinical diagnosis largely depends on visual inspection and dermatoscopy, but these methods are inherently subjective and often lack sensitivity for detecting early microlesions or reliably distinguishing melanoma from benign pigmented nevi (3, 4). For prognosis assessment, commonly used parameters such as Breslow depth and lymph node status reflect only local tumor characteristics and fail to capture the tumor’s molecular behavior, making accurate prediction of long-term survival difficult (5). Furthermore, approximately 30%-50% of patients with advanced melanoma develop resistance to targeted therapies or immune checkpoint inhibitors (ICIs), and effective biomarkers to predict treatment response are still lacking in clinical practice (6, 7). Thus, there is an urgent need to identify highly sensitive and robust molecular biomarkers for early diagnosis, accurate prognosis, and treatment response prediction in melanoma.

**Figure 1:**
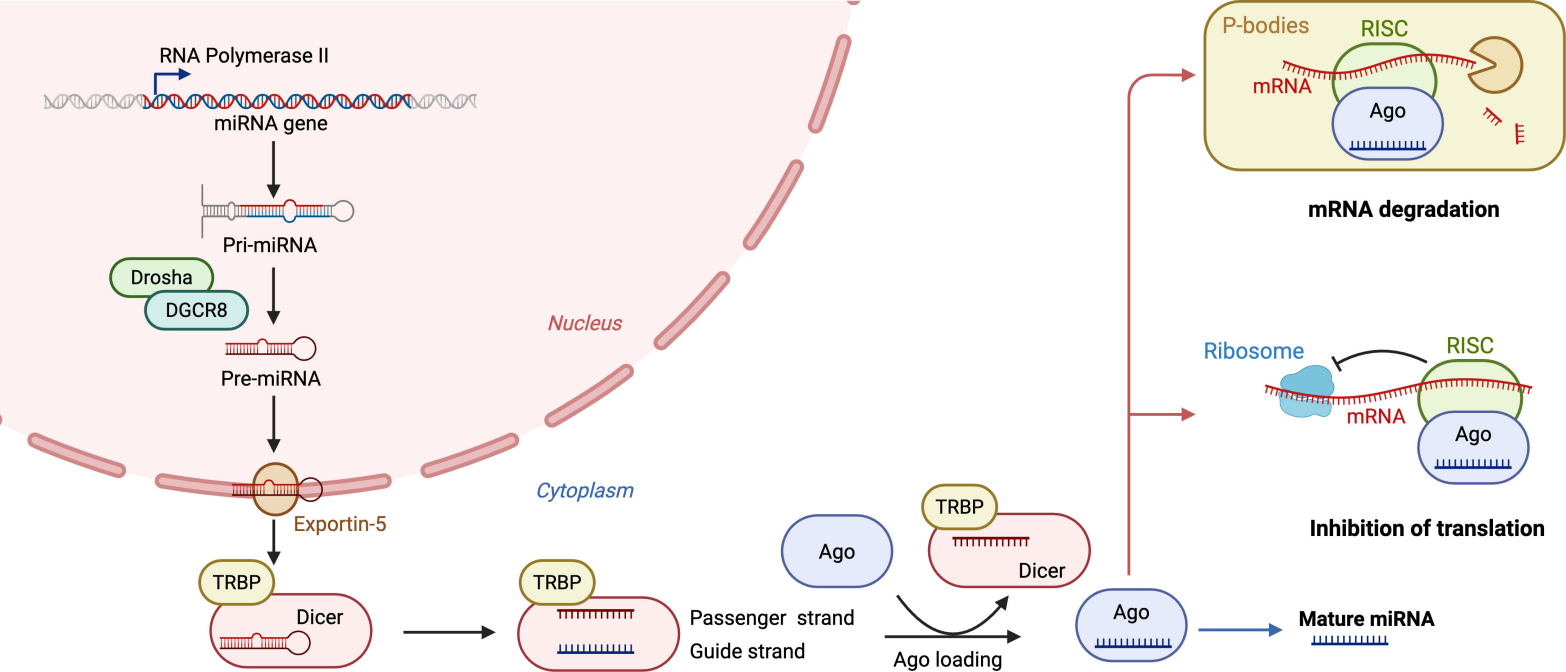
Schematic diagram of canonical miRNA biosynthesis and post-transcriptional regulatory mechanisms. This figure illustrates the canonical biogenesis pathway of miRNAs and their post-transcriptional regulation of target genes. In the nucleus, pri-miRNAs are processed into pre-miRNAs by the Drosha–DGCR8 microprocessor complex and then exported to the cytoplasm by Exportin-5. In the cytoplasm, pre-miRNAs are further cleaved by the Dicer–TRBP complex to generate miRNA duplexes. The guide strand is incorporated into the RISC with Ago proteins, whereas the passenger strand is degraded. Upon binding to target mRNA, the mature miRNA–RISC complex induces mRNA degradation or translational repression. miRNA, microRNA; pri-miRNA, primary microRNA; pre-miRNA, precursor microRNA; DGCR8, DiGeorge syndrome critical region 8; TRBP, TAR RNA-binding protein; Ago, Argonaute; RISC, RNA-induced silencing complex.

MicroRNAs are a class of small non-coding RNAs, approximately 18–25 nucleotides in length, that mainly exert biological functions by post-transcriptionally regulating target gene expression. Their canonical biogenesis and mechanisms of action are illustrated in **Figure 1**. Owing to their high sensitivity, relative stability, and non-invasive detectability, miRNAs have been considered promising diagnostic biomarkers. In melanoma, miRNAs can function either as oncogenic factors or tumor suppressors, precisely regulating downstream signaling networks that drive tumor progression (8). Compared with other molecular biomarkers, such as mRNAs and proteins, miRNAs offer significant advantages for clinical translation. Their short length, together with protection by EVs or protein complexes, confers remarkable stability in body fluids such as blood and urine, enabling resistance to degradation (9). In addition, miRNAs can simultaneously regulate genes involved in multiple signaling pathways, thereby providing a more comprehensive view of tumor biology. These features make miRNAs promising candidate biomarkers for non-invasive liquid biopsy in melanoma.

During melanoma development and progression, miRNA expression profiles often exhibit dynamic and stage-related changes. For example, Mueller et al. identified 49 miRNAs that were consistently upregulated during the transformation of normal melanocytes to metastatic cell lines using microarray analysis. Among these, six miRNAs, including miR-133a and miR-199b, demonstrated more than three-fold increases in expression, while miR-190 was progressively downregulated (10). Expression profile differences can also distinguish melanoma subtypes, such as acral versus mucosal melanoma (MucM), and provide a foundation for molecular subtyping (11). These expression abnormalities arise through various mechanisms, including genomic alterations (e.g., copy number variations), epigenetic modifications (e.g., promoter hypermethylation), and the dysregulation of miRNA processing pathways. For instance, miR-34a is frequently silenced in melanoma due to promoter methylation, which releases its target gene, zinc finger E-box binding homeobox 1 (*ZEB1*), thereby promoting tumor metastasis (12). Conversely, overexpression of the processing enzyme Dicer is also common in melanoma and correlates with increased tumor invasiveness (13).

Beyond these cell-intrinsic functions, miRNAs are increasingly recognized as key regulators of the TIME. They shape anti-tumor immune responses by influencing immune checkpoint expression, modulating immune cell recruitment and function, and facilitating intercellular communication via EVs (14, 15). This immunomodulatory capacity adds another layer of complexity, positioning miRNAs not only as direct regulators of tumor cells but also as potential biomarkers for immune status and immunotherapy response.

In summary, miRNAs exhibit significant potential for clinical application in melanoma. The following sections will systematically discuss the current research status and application prospects of miRNAs as biomarkers in three key areas: early diagnosis, prognostic stratification, and treatment response prediction of melanoma. We will then provide a comprehensive overview of their immunomodulatory functions, with an emphasis on their relevance to the TIME and their emerging role in guiding immunotherapy.

## 2 MicroRNAs as diagnostic biomarkers in melanoma

Optimal diagnostic markers are characterized by high sensitivity, relative stability, non-invasiveness, and ease of detection. MicroRNAs offer considerable advantages in these respects. According to sample source, diagnostic miRNA biomarkers can be broadly grouped into tissue-derived, blood-derived, and EVs-derived categories, each of which includes both individual miRNAs and multi-miRNA panels with translational potential.

### 2.1 Tissue miRNA diagnostic markers

The miRNA expression profile in tissue samples directly reflects the molecular characteristics of tumor cells, providing valuable information for cases where pathological diagnosis is challenging. Babapoor et al. used *in situ* hybridization to show that miR-211 is highly expressed in normal and atypical nevus tissues but significantly downregulated in melanoma tissues, with a sensitivity of 90% and specificity of 86.2% for distinguishing melanoma from pigmented nevi (16). Another study confirmed that miR-203 expression is reduced in melanoma tissue and correlates with tumor metastasis, making it a potential indicator for assessing metastatic potential (17). Furthermore, miR-378 is overexpressed in melanoma tissues, where it activates the Wnt/β-catenin pathway by inhibiting forkhead box N3 (*FOXN3*). Its expression level is positively correlated with tumor invasion depth, making it a potential biomarker for assessing tumor aggressiveness (18). Beyond individual tissue-derived miRNAs, tissue-based multi-miRNA panels may further enhance diagnostic accuracy. For instance, a study developed a diagnostic signature panel comprising five miRNAs, including miR-31-5p, miR-21-5p, miR-211-5p, miR-125a/b-5p and miR-100-5p, and showed their capability of distinguishing clinically challenging nevi from melanoma (19).

### 2.2 Blood miRNA diagnostic markers

Circulating miRNAs in the blood (plasma/serum) are safeguarded by exosomes or protein complexes, thereby exhibiting high stability and potential as non-invasive diagnostic tools. Among blood-based biomarkers, multi-miRNA panels have shown particularly strong promise for the non-invasive diagnosis of melanoma. For instance, Fogli et al. identified that miR-150-5p and miR-149-3p were upregulated, whereas miR-193a-3p was downregulated in the plasma of melanoma patients. The combined detection of these three miRNAs achieved a sensitivity of 94.8% and specificity of 83.9% in differentiating patients from healthy controls (20). Solé et al. reported significantly reduced plasma expression levels of miR-134-5p and miR-320a-3p in stage 0 melanoma patients, with diagnostic specificity and sensitivity of 96% and 90%, respectively, indicating their potential for early screening (21). In addition to these panels, Armand-Labit et al. identified a circulating two-miRNA signature consisting of miR-1246 and miR-185 that distinguished patients with metastatic cutaneous melanoma from healthy donors, achieving a sensitivity of 90.5% and a specificity of 89.1%. These findings further support the diagnostic potential of circulating miRNA profiles in melanoma (22).

Certain miRNAs can also differentiate melanoma from other skin lesions, predominantly characterized by nevi. For example, the MEL-38 miRNA panel, developed by van Laar et al., comprises 38 differentially expressed miRNAs. Through large-scale multicenter validation via liquid biopsy, MEL-38 demonstrated excellent performance, with a sensitivity of 93% and specificity of 98% in a multicenter validation involving 582 plasma samples (23). Another study identified six miRNAs, including miR-5694 and miR-6796-3p, that specifically recognize melanoma brain metastases, effectively distinguishing them from other types of brain metastases and glioblastoma (24).

### 2.3 Extracellular vesicles miRNA diagnostic markers

Exosomes, a subtype of EVs, range from 30–150 nm in diameter. They effectively transport molecules, such as proteins and nucleic acids (e.g., miRNAs and DNA), originating from parent melanoma cells, and subsequently release them into body fluids, such as plasma. The miRNAs contained within these exosomes not only mirror the real-time molecular characteristics of tumors but also provide insights into the state of the tumor microenvironment (25). Urinary extracellular miRNAs present significant potential for therapeutic monitoring because of their non-invasive and readily accessible characteristics. Research indicates that in melanoma patients undergoing immunotherapy, the expression levels of peripheral blood extracellular miRNAs, specifically miR-155 and miR-125b-5p, decrease, and these alterations are positively correlated with treatment response (26). Furthermore, research has demonstrated that microarray analysis can identify 55 differentially expressed miRNAs in plasma exosomes from patients with melanoma. Another study reported reduced expression of exosomal miR-1180-3p in the plasma of patients with melanoma, which facilitates tumor cell migration by targeting the ST3 beta-galactoside alpha-2,3-sialyltransferase 4 (*ST3GAL4*) gene. This miRNA exhibited diagnostic sensitivity and specificity of 82.1% and 78.6%, respectively (27). Similarly, Sabato et al. identified a plasma EV-associated four-miRNA signature consisting of miR-412-3p, miR-507, miR-1203, and miR-362-3p, which showed robust diagnostic performance across three independent cohorts (28).

**Figure 2:**
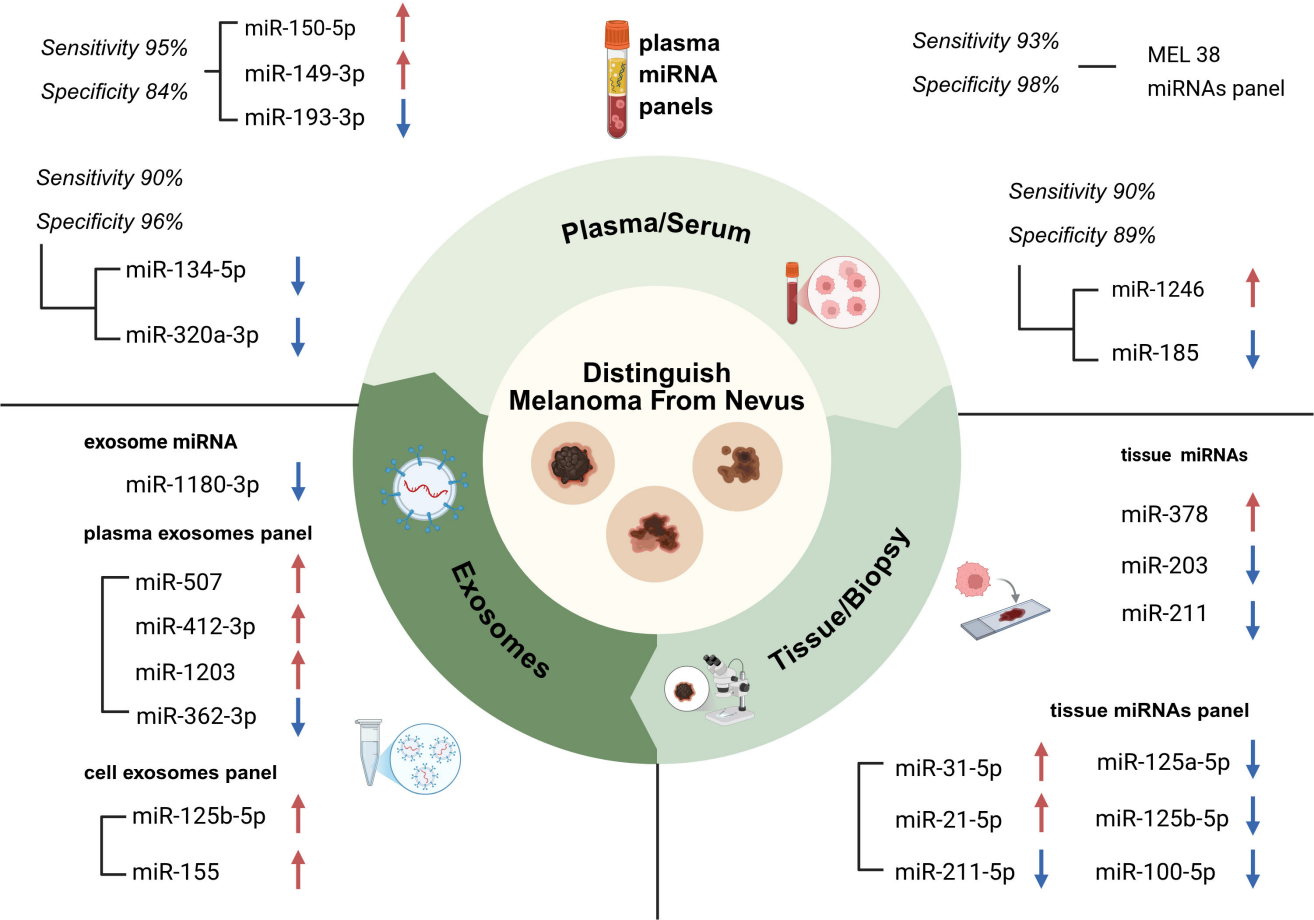
Overview of melanoma-related miRNA diagnostic biomarkers. This figure summarizes representative miRNA biomarkers for melanoma diagnosis according to sample source, including tissue/biopsy-derived miRNAs and multi-miRNA panels, plasma/serum-derived circulating miRNAs and multi-miRNA panels, and EV-derived miRNAs and multi-miRNA panels. Diagnostic performance metrics, including sensitivity and specificity, are annotated for selected biomarkers. Red upward arrows and blue downward arrows indicate the relative upregulation or downregulation, respectively, in melanoma samples compared with control samples. miRNA, microRNA; EV, extracellular vesicle.

In conclusion, miRNAs have emerged as essential molecular tools for liquid biopsy and histopathological auxiliary diagnosis of melanoma due to their tissue specificity, high stability in body fluids and enrichment in EVs. **Figure 2** comprehensively summarizes diagnostic miRNA biomarkers associated with melanoma categorized by sample source. With continuous advances in detection technologies and ongoing validation in prospective clinical cohorts, miRNA biomarkers hold significant promise for further integration into precision melanoma diagnostics and therapeutics in the future. They offer robust support for the early detection, subtyping, and personalized management of patients.

**Table 1** summarizes the biological functions and clinical applications of upregulated and downregulated miRNAs as diagnostic markers.

**Table 1:** Summary of miRNA biomarkers for melanoma diagnosis.

miRNA	Sample	Expression pattern	Biological function	Clinical application direction	References
miR-211	Tissue	Downregulated	Associated with melanoma metastasis	Distinguishing melanoma from benign nevi (sensitivity 90%, specificity 86.2%)	(16)
miR-203	Tissue	Downregulated	Associated with melanoma metastasis	Potential biomarker for assessing melanoma metastatic potential	(17)
miR-378	Tissue	Upregulated	Inhibits *FOXN3* to activate the Wnt/β-catenin pathway, promoting proliferation and migration	Potential biomarker for assessing tumor invasiveness	(18)
miR-31-5p	Tissue	Upregulated	Associated with the differentiation of melanocytes, the quiescent state of nevi, and the malignant transformation of melanoma.	Distinguishing melanoma from benign nevi in diagnostically challenging cases	(19)
miR-21-5p		Upregulated			
miR-211-5p		Downregulated			
miR-125a/b-5p		Downregulated			
miR-100-5p		Downregulated			
miR-150-5p	Plasma	Upregulated	Inhibits apoptosis and promotes proliferation	Combined diagnosis of melanoma (sensitivity 94.8%, specificity 83.9%)	(20)
miR-149-3p		Upregulated			
miR-193a-3p		Downregulated			
miR-134-5p	Plasma	Downregulated	Inhibits tumor proliferation and invasion, promotes apoptosis	Combined diagnosis of stage 0/I/II melanoma (specificity 96%, sensitivity 90%)	(21)
miR-320a-3p	Plasma	Downregulated			
miR-1246	Plasma	Upregulated	Associated with metastatic melanoma	Combined diagnosis of metastatic cutaneous melanoma (sensitivity 90.5%, specificity 89.1%)	(22)
miR-185		Downregulated			
MEL-38 (including miR-424-5p, miR-5481, miR-34-5p, etc.)	Plasma	–	miRNA signature panel	Combined diagnosis of stage 0/I/II/III/IV melanoma (sensitivity 93%, specificity 98%)	(23)
miR-5694	Cerebrospinal fluid	Upregulated	Associated with brain metastasis-related regulation	Diagnosis of melanoma brain metastasis	(24)
miR-6796-3p		Upregulated			
miR-362-3p		Upregulated			
miR-125b-5p	Melanoma cell exosomes	Upregulated	Associated with tumor progression	Combined diagnosis of melanoma	(26)
miR-155	Melanoma cell exosomes	Upregulated	Associated with tumor progression	Combined diagnosis of melanoma	(26)
miR-1180-3p	Plasma Exosomes	Downregulated	Targets *ST3GAL4*, inhibiting tumor-cell migration	Melanoma diagnosis (sensitivity 82.1%, specificity 78.6%)	(27)
miR-412-3p	Exosomes	Upregulated	miRNA signature panel	Combined diagnosis of metastatic melanoma	(28)
miR-507		Upregulated			
miR-1203		Upregulated			
miR-362-3p		Downregulated			

Upregulated or downregulated indicates higher or lower miRNA expression in melanoma samples relative to the corresponding control group.

## 3 MicroRNAs as prognostic biomarkers in melanoma

Prognostic biomarkers are instrumental in predicting patient survival, tumor recurrence, and metastatic risk. In melanoma, the following sections summarize miRNA expression alterations associated with these three major prognostic outcomes. Each section incorporates evidence derived from different sample sources and includes both individual miRNAs and multi-miRNA panels.

### 3.1 MicroRNAs associated with survival prognosis

Numerous studies have established a significant correlation between the expression levels of specific miRNAs and overall survival (OS), disease-free survival (DFS), and distant metastasis-free survival (DMFS) in patients with melanoma. For example, miR-137 is downregulated in cutaneous melanoma (CM) tissues, and its reduced expression is associated with a diminished 5-year OS. Multivariate analysis identified miR-137 as an independent prognostic factor (29). Exosomal miRNAs may also have prognostic relevance. Melanoma cell-derived exosomal miR-424-5p is upregulated and promotes endothelial proliferation, migration, and angiogenesis by directly targeting large tumor suppressor kinase 2 (*LATS2*), thereby facilitating tumor growth *in vivo*. Although current evidence is limited to experimental models, this miRNA may represent a progression-related exosomal biomarker (30). Serum miRNAs exhibit significant potential for non-invasive prognostic evaluation. A study involving 120 melanoma patients identified a marked reduction in serum miR-16 levels, which further diminished with increasing tumor thickness, ulceration, and American Joint Committee on Cancer (AJCC) stage progression. Low serum miR-16 was found to be an independent adverse predictor of OS in multivariate Cox analysis. Mechanistic investigations suggest that miR-16 suppresses cell proliferation and induces apoptosis by downregulating the apoptosis regulator B-cell lymphoma 2 (*BCL2*) and cyclin D1 (*CCND1*) (31–33). Another study demonstrated that serum miR-23a is significantly downregulated in metastatic melanoma, with low levels independently associated with poor OS. The underlying mechanism involves inhibition of autophagy by targeting autophagy related 12 (*ATG12*), thereby reducing tumor cell invasion and metastasis (34).

MicroRNA expression patterns across various molecular subtypes have prognostic significance. Studies have indicated that miR-204 is abundantly expressed in normal melanocytes but is markedly downregulated in melanoma tissues, particularly within the *NRAS* wild-type subtype. Its diminished expression is independently correlated with increased Breslow thickness and reduced OS. Restoration of miR-204 expression inhibits the proliferation of *BRAF*/*CDKN2A* wild-type cell lines (35). In contrast, miR-211 is generally downregulated in melanoma, with the lowest expression observed in the *BRAF*/*CDKN2A* double-mutant subgroup of the TCGA cohort. This downregulation is positively correlated with Breslow thickness, suggesting that miR-211 may serve as a marker for a more aggressive molecular subtype (36).

Beyond individual prognostic miRNAs, multi-miRNA panels have been developed to further improve predictive accuracy. One study screened 80 upregulated and 105 downregulated miRNAs to establish a five-miRNA signature, including miR-25, miR-204, miR-211, miR-510, and miR-513c. This signature is correlated with tumor T stage and Breslow depth, effectively predicting patient survival (37). Another study identified significant associations between distinct miRNA expression profiles in melanoma and patient survival outcomes. The prognostic panel, comprising miR-200a-3p, miR-335-5p, miR-451a, and miR-29b-3p, offers specific predictive value across various disease stages and treatment responses (38).

**Table 2** summarizes melanoma-associated miRNAs related to survival prognosis.

**Table 2:** Summary of miRNA biomarkers associated with melanoma prognosis (survival outcomes).

miRNA	Sample	Expression pattern	Biological function	Clinical application direction	References
miR-137	Tissue	Downregulated	Targets *EZH2* to block the Wnt/β-catenin pathway, inhibiting tumor progression.	Low expression is associated with poor OS.	(29)
miR-424-5p	Exosomes	Upregulated	Associated with tumor metastatic status.	Potential progression biomarker	(30)
miR-16	Serum	Downregulated	Targets *BCL2* and *CCND1*, inhibiting cell proliferation and inducing apoptosis.	Low expression independently correlates with poor OS.	(31–33)
miR-23a	Serum	Downregulated	Targets *ATG12*, inhibiting autophagy, and weakening tumor cell invasion and metastatic capacity.	Low expression is independently associated with poor OS.	(34)
miR-204	Tissue	Downregulated	Inhibits the growth of *BRAF* wild-type cell lines.	Low expression is independently associated with greater Breslow thickness and poorer OS.	(35)
miR-211	Tissue	Downregulated	Targets *PDK4*, inhibiting melanoma progression.	Low expression correlates with greater Breslow thickness, indicating a more aggressive molecular subtype.	(36)
miR-25	Tissue	Upregulated	Target genes may participate in the PI3K/AKT pathway, ubiquitin-mediated proteolysis, and focal adhesion.	Prognostic biomarker	(37)
miR-204		Downregulated			
miR-211		Upregulated			
miR-510		Downregulated			
miR-513c		Downregulated			
miR-200a-3p	Tissue	Upregulated	miRNA signature panel	Prognostic biomarker	(38)
miR-335-5p		Upregulated			
miR-451a		Downregulated			
miR-29b-3p		Downregulated			

Upregulation or downregulation indicates higher or lower miRNA expression in the poor-OS group relative to the favorable-OS group.

### 3.2 MicroRNAs associated with tumor metastasis

Metastasis is the principal cause of mortality in patients with melanoma. MicroRNAs modulate the metastatic potential of tumors by regulating processes such as epithelial-mesenchymal transition (EMT) and angiogenesis (39). Mechanistically, miR-10b promotes melanoma cell invasion and metastasis by targeting homeobox D10 (*HOXD10*), leading to upregulation of ras homolog family member C (*RHOC*), a small GTPase involved in cytoskeletal reorganization and cell motility (40). Detailed mechanistic studies have revealed complex regulatory networks governing metastatic progression. miR-519d is markedly upregulated in metastatic melanoma, promoting lung metastasis by inhibiting EPH receptor A4 (*EPHA4*), which leads to activation of the extracellular signal-regulated kinase 1/2 (ERK1/2) pathway and facilitation of EMT. Patients with metastatic lesions characterized by low *EPHA4* expression exhibit significantly reduced OS, providing a mechanistic link between this miRNA axis and clinical outcomes (41).

In recent years, the role of extracellular miRNAs in predicting metastasis has attracted considerable scholarly attention. For instance, miR-146a-5p, which is elevated in melanoma brain metastasis (MBM) cells and their exosomes, promotes metastasis by targeting NUMB endocytic adaptor protein (*NUMB*) in astrocytes and activating Notch signaling, leading to the release of tumor-promoting cytokines [IL-6, IL-8, C-C motif chemokine ligand 2 (CCL2), C-X-C motif chemokine ligand 1 (CXCL1)] that reshape the brain metastatic niche (42). Research shows that miR-125b suppresses melanoma proliferation and metastasis by targeting mitogen-activated protein kinase kinase kinase 11 (*MAP3K11*) and Jun proto-oncogene, AP-1 transcription factor subunit (*JUN*), with its reduced expression correlating with lymph node metastasis and poor prognosis (43–45). Additionally, low serum miR-206 expression is associated with an elevated risk of metastasis and decreased 5-year OS (46). Stem cell-associated miRNAs also play a regulatory role in metastasis. Exosomes derived from melanoma stem cells (MSCs) are enriched in miR-592, which can be internalized by cells with low metastatic potential. This process inhibits protein tyrosine phosphatase non-receptor type 7 (*PTPN7*), thereby activating the MAPK/ERK pathway, which in turn enhances cell migration, invasion, and the capacity for lung metastasis colonization (47). Similarly, miR-487a-5p present in highly metastatic exosomes facilitates the progression of low-metastatic cells to multi-organ metastasis by downregulating nudix hydrolase 21 (*NUDT21*) and promoting glycolysis (48).

**Table 3** summarizes miRNAs associated with melanoma metastasis progression.

**Table 3:** Summary of miRNA biomarkers associated with melanoma prognosis (tumor metastasis).

miRNA	Sample	Expression pattern	Biological function	Clinical application direction	References
miR-10b	Tissue	Upregulated	Targets *HOXD10* to increase *RHOC* expression, enhancing invasion and metastasis	Tumor metastasis indicator	(40)
miR-519d	Tissue Cells	Upregulated	Targets *EPHA4* to activate the ERK1/2 pathway, enhancing invasion and metastasis	Low *EPHA4* expression is associated with shorter OS	(41)
miR-146a-5p	Cells Exosomes	Upregulated	Inhibits *NUMB* to activate Notch signaling, reshaping the brain metastasis microenvironment	Brain metastasis indicator	(42)
miR-125b	Tissue Exosomes	Downregulated	Targets *MAP3K11* and *JUN*, inhibiting proliferation and metastasis	Low expression is associated with lymph node metastasis and poor prognosis	(43)
miR-206	Serum	Downregulated	Targets *CDK4* and *CCND1*	Low expression is associated with increased metastasis risk and shorter OS	(46)
miR-592	Exosomes	Upregulated	Targets *PTPN7* to activate the MAPK/ERK pathway, enhancing invasion and metastasis	Lung metastasis indicator	(47)
miR-487a-5p	Exosomes	Upregulated	Targets *NUDT21*, promoting glycolysis-related processes	Induces progression of low-metastatic cells toward multi-organ metastasis	(48)

Upregulated or downregulated indicates higher or lower miRNA expression in metastatic melanoma samples relative to non-metastatic, low-metastatic, or corresponding reference controls.

### 3.3 MicroRNAs associated with recurrence

Tumor recurrence remains a major challenge in melanoma treatment, and miRNAs may serve as dynamic biomarkers for predicting recurrence. Certain miRNAs influence recurrence by modulating cell cycle progression and stem cell properties. For example, miR-222 enhances melanoma proliferation and stem cell-like characteristics by targeting cyclin dependent kinase inhibitor 1B (*CDKN1B*), with its overexpression showing a positive correlation with recurrence (49). In contrast, miR-34a inhibits EMT by suppressing *ZEB1*, and patients with low miR-34a expression are at an increased risk of postoperative recurrence (12). Mechanistically, miR-101-3p is downregulated in melanoma and promotes early tumor progression by targeting lamin B1 (*LMNB1*), a nuclear lamin protein involved in maintaining nuclear structure and genomic stability. Downregulation of *LMNB1* leads to genomic instability, facilitating malignant transformation and recurrence (50). Furthermore, miR-211-5p, which is abundantly secreted by metastatic exosomes, suppresses G protein subunit alpha 15 (*GNA15*), reduces anti-tumor immune infiltration, and inhibits pyroptosis—a form of immunogenic cell death—thereby promoting immune evasion and malignant progression in melanoma (51).

Beyond individual recurrence-associated miRNAs, panel-based approaches have also shown added value for risk stratification. Serum-based investigations have identified a four-miRNA signature consisting of miR-150, miR-30d, miR-15b, and miR-425. When integrated with clinical staging, this signature markedly enhances risk stratification for RFS and OS (52). At the tissue level, the combined expression levels of miR-21-5p and miR-146a-5p exhibit a strong correlation with Breslow thickness. Patients with elevated expression experience shorter recurrence intervals and diminished OS, offering superior prognostic stratification compared to tumor thickness alone (53).

**Table 4** summarizes miRNAs associated with melanoma recurrence.

**Table 4:** Summary of miRNA biomarkers associated with melanoma prognosis (recurrence).

miRNA	Sample	Expression pattern	Biological function	Clinical application direction	References
miR-222	Tissue Exosomes	Upregulated	Targets *CDKN1B*, promoting proliferation and stem cell-like properties and regulating the PI3K/AKT pathway	High expression is associated with recurrence risk	(49)
miR-34a	Cells	Downregulated	Inhibits *ZEB1* to impede EMT	Low expression is associated with recurrence risk	(12)
miR-101-3p	Cells	Downregulated	Inhibits *LMNB1* to affect genomic stability	Tumor progression and recurrence risk indicator	(50)
miR-211-5p	EVs derived from highly metastatic cells	Upregulated	Inhibits *GNA15*, reducing anti-tumor immune infiltration, and suppressing pyroptosis	Tumor progression and recurrence risk indicator	(51)
miR-150 miR-15b miR-30d miR-425	Serum	Upregulated	miRNA signature panel	Tumor recurrence risk indicator	(52)
miR-146a-5p	Tissue	Upregulated	Inhibits *NUMB* to activate Notch signaling	High expression is associated with shorter time to recurrence and poorer OS	(53)
miR-21-5p	Tissue	Upregulated	Targets *PDCD4*, *PTEN*, and *BCL2*, promoting melanoma growth and progression.	High expression is associated with shorter time to recurrence and poorer OS	(53)

Upregulated or downregulated indicates higher or lower miRNA expression in the recurrence group relative to the non-recurrence, low-risk or corresponding reference group.

**Figure 3:**
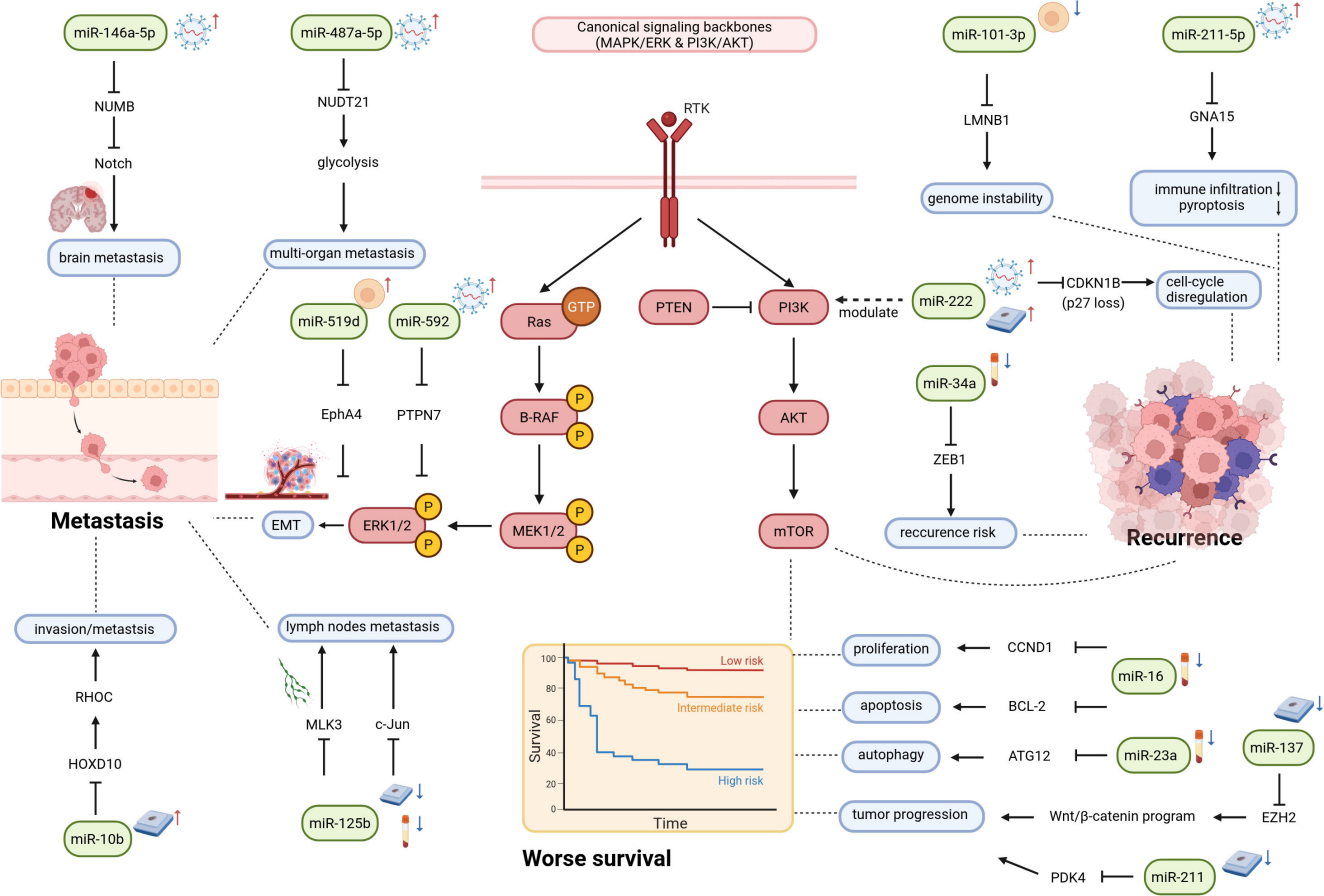
MiRNA biomarkers for melanoma prognosis: stratification of metastasis, recurrence, and survival risk. This figure summarizes representative miRNA–target gene interactions associated with melanoma prognosis. It is organized around three prognostic dimensions: metastasis, recurrence, and survival. The major signaling modules include the MAPK/ERK pathway, which is mainly associated with invasion and metastasis, and the PI3K/AKT pathway, which is mainly associated with survival and recurrence-related phenotypes. Red upward arrows and blue downward arrows indicate miRNA upregulation and downregulation, respectively, in patients with poor prognosis relative to those with favorable prognosis. Dashed arrows indicate associations with specific clinical outcomes. miRNA, microRNA; MAPK, mitogen-activated protein kinase; ERK, extracellular signal-regulated kinase; PI3K, phosphatidylinositol 3-kinase; AKT, protein kinase B; RTK, receptor tyrosine kinase; PTEN, phosphatase and tensin homolog; mTOR, mechanistic target of rapamycin; EMT, epithelial–mesenchymal transition.

In conclusion, miRNAs have significant clinical utility as prognostic biomarkers for melanoma. In terms of survival prognosis, miRNAs derived from tissues and serum, as well as multi-miRNA signature panels, are effective in predicting OS and DFS. Certain miRNAs demonstrate stratified prognostic significance across various molecular subtypes. In the context of metastasis risk assessment, miRNAs modulate the metastatic potential of tumors by regulating critical pathways. Notably, EV-derived miRNAs play a pivotal role in mediating organ-specific metastasis and remodeling the pre-metastatic microenvironment, offering novel non-invasive indicators for the early detection of brain, lung, and multi-organ metastases. For recurrence monitoring, circulating miRNAs and multi-miRNA combinations facilitate dynamic risk assessment and early detection of recurrence, exhibiting superior predictive performance compared with traditional clinical-pathological indicators. These findings enhance our understanding of the mechanisms underlying melanoma progression, providing a crucial foundation for the development of multidimensional prognostic prediction models, guiding personalized follow-up, and informing targeted interventions against metastasis and recurrence (**Figure 3**).

## 4 MicroRNAs as therapeutic predictive biomarkers in melanoma

The essence of precision medicine is the selection of appropriate treatment regimens based on individual molecular characteristics of the disease. In recent years, non-coding RNAs, particularly miRNAs, have shown considerable potential for predicting the efficacy of chemotherapy, targeted therapy, and immunotherapy. They achieve this by regulating key therapeutic targets and pathways related to drug resistance, thereby providing new molecular evidence for personalized melanoma treatment.

### 4.1 Chemotherapy sensitivity prediction

Chemoresistance is a major obstacle to successful treatment outcomes in melanoma. Research has demonstrated that variations in the expression of specific miRNAs are closely associated with the sensitivity of melanoma cells to chemotherapeutic agents, indicating their predictive potential. For example, studies show that miR-181a/b can enhance cellular sensitivity to chemotherapy drugs. Its mechanism involves targeting mitochondrial transcription factor A (*TFAM*) to suppress mitochondrial function. Clinical data reveal that patients exhibiting high miR-181a/b expression experience significantly improved chemotherapy response rates compared to those with lower expression levels (54). Additionally, miR-34a promotes apoptosis by targeting the anti-apoptotic protein BCL2, and its elevated expression levels are positively correlated with the sensitivity of melanoma cells to cisplatin (55). Notably, recent studies have revealed that chemotherapy efficacy is also influenced by the TIME, and miRNAs play a bridging role. For instance, Lapkina et al. demonstrated that miR-204-5p modulates the response of melanoma cells to dacarbazine through pathways that are intimately linked to immune microenvironment remodeling, specifically the nuclear factor kappa B (NF-κB) pathway and IL-17 signaling, highlighting how miRNAs can integrate chemotherapy stress signals with immune regulation (56). These findings suggest that specific miRNA expression profiles may serve as potential biomarkers for predicting chemotherapy sensitivity, thereby informing clinical drug selection.

### 4.2 Targeted therapy sensitivity prediction

Targeted therapy has emerged as a pivotal treatment strategy for *BRAF*-mutant melanoma; however, the issue of acquired resistance persists as a substantial obstacle. Research indicates that variations in the expression of multiple miRNAs are intricately linked to treatment effectiveness and the emergence of resistance.

#### 4.2.1 MicroRNAs predicting efficacy and sensitivity

In the context of predicting treatment efficacy, elevated expression of specific miRNAs is associated with an enhanced therapeutic response. For example, miR-524-5p increases tumor cell sensitivity to *BRAF* inhibitors by inhibiting the MAPK/ERK signaling pathway. Patients exhibiting high pre-treatment tissue expression of miR-524-5p experienced significantly extended progression-free survival (PFS). Meanwhile, research has indicated decreased miR-524-5p expression in vemurafenib-resistant cells and tissues, whereas patients who achieved complete or sustained partial remission showed notably higher pre-treatment tissue levels of miR-524-5p (57). Another study involving 70 patients with advanced *BRAF*-mutant melanoma found that elevated baseline serum levels of miR-579-3p and reduced baseline levels of miR-4488 prior to treatment were correlated with prolonged PFS following *BRAF* inhibitor therapy (58). Similarly, miR-876-3p, which is downregulated in melanoma, enhances tumor cell sensitivity to vemurafenib in preclinical models by directly targeting *MAPK1* to inhibit ERK signaling pathways, thereby indirectly demonstrating a positive correlation between its high expression and treatment efficacy (59).

#### 4.2.2 MicroRNAs predicting resistance risk

In the context of resistance prediction, the upregulation of specific miRNAs is associated with resistance to targeted therapy. Detailed mechanistic studies have elucidated multiple pathways through which miRNAs confer drug resistance. In vemurafenib-resistant models, the drug induces demethylation of the *MIR152* promoter, resulting in its upregulation. This process suppresses thioredoxin-interacting protein (*TXNIP*), thereby driving tumor cells toward a slow-cycling, highly invasive phenotype and enhancing their metastatic potential (60). Similarly, in cells with acquired resistance to vemurafenib, there is a rapid increase in the expression of miR-204-5p and miR-211-5p, which promote drug tolerance; inhibition of these miRNAs partially restores cellular sensitivity (61). In MAPK inhibitor-resistant models, miR-4443 and miR-4488 were upregulated. These miRNAs target the downregulation of the intermediate filament protein Nestin (*NES*), leading to cytoskeletal remodeling and enhanced migration and invasion. Clinical correlation analysis has revealed that patients with higher *NES* protein levels in pre-treatment biopsy tissues exhibit longer PFS, suggesting that the miR-4443/4488-*NES* axis is correlated with drug resistance (62). Another study identified an alternative resistance pathway: in dabrafenib-resistant models, the downregulation of miR-126-3p resulted in the upregulation of its target genes ADAM metallopeptidase domain 9 (*ADAM9*) and vascular endothelial growth factor A (*VEGFA*), thereby promoting proliferation, invasion, and resistance. Restoration of miR-126-3p expression reverses these phenotypes (63, 64).

Furthermore, inhibition of the MAPK pathway itself induced the upregulation of miR-143-3p and miR-145-5p. These miRNAs promote tumor cell dedifferentiation towards a mesenchymal phenotype characterized by high AXL receptor tyrosine kinase (AXL) expression and low microphthalmia-associated transcription factor (MITF) expression through the downregulation of fascin actin-bundling protein 1 (*FSCN1*), thereby conferring drug resistance (65). Another study elucidated dynamic changes in the positive feedback loop between the transcription factor MITF and miR-579-3p during drug resistance. This loop induces growth arrest in the early treatment phase; however, during acquired resistance, their expression is synchronously downregulated while AXL is upregulated. Dysregulation of this loop correlates with the recurrence of clinical drug resistance (66).

**Figure 4:**
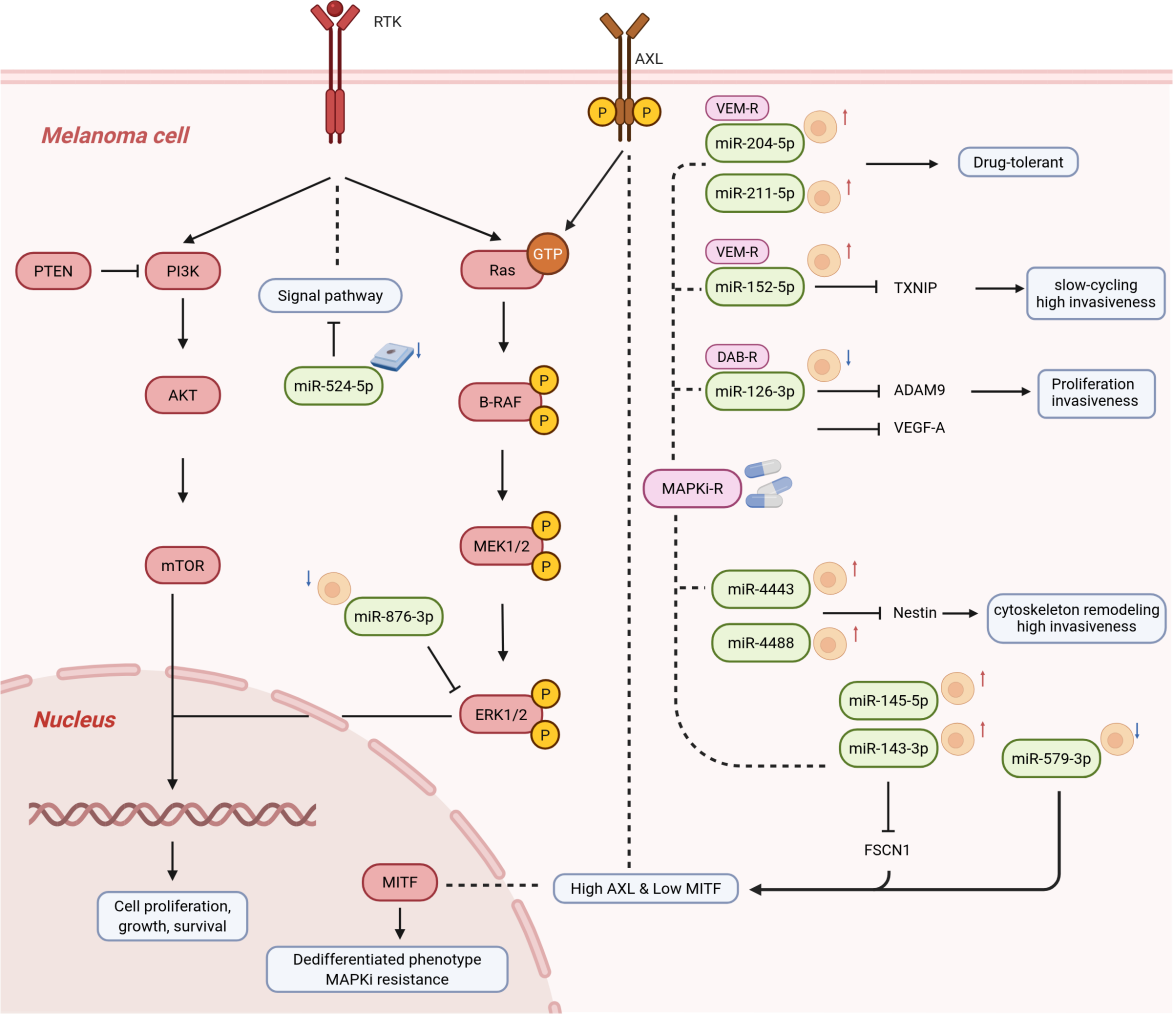
Overview of miRNA biomarkers for predicting response to targeted therapy in melanoma. This figure summarizes representative miRNA signatures associated with response or resistance to targeted therapy in melanoma. The major signaling modules include the MAPK/ERK pathway, a central driver of *BRAF*-mutant melanoma and a key determinant of sensitivity to *BRAF* inhibition; the PI3K/AKT/mTOR axis, which supports survival and compensatory resistance; and the AXL-high/MITF-low dedifferentiation program, which reflects phenotype switching associated with adaptive MAPK inhibitor resistance. The figure also highlights downstream phenotypes, including slow-cycling persistence, invasiveness, and cytoskeletal remodeling. Sample sources include tumor tissue, serum/plasma-derived circulating miRNAs, EV-derived miRNAs, and cell-based or *in vitro* resistance models. Red upward arrows and blue downward arrows indicate miRNA upregulation and downregulation, respectively, in poor responders or resistant groups relative to responders or sensitive groups. miRNA, microRNA; MAPK, mitogen-activated protein kinase; ERK, extracellular signal-regulated kinase; PI3K, phosphatidylinositol 3-kinase; AKT, protein kinase B; mTOR, mechanistic target of rapamycin; RTK, receptor tyrosine kinase; MITF, microphthalmia-associated transcription factor; AXL, AXL receptor tyrosine kinase; VEM-R, vemurafenib-resistant; DAB-R, dabrafenib-resistant; MAPKi-R, MAPK inhibitor-resistant.

MicroRNA signatures associated with targeted therapy responses can be categorized based on their expression patterns in responders versus non-responders, downstream target genes and signaling pathways, and resultant cellular phenotypes. The sample sources of these predictive biomarkers are diverse, including tumor tissue/baseline biopsies, serum/plasma circulating miRNAs, exosomal/EV miRNAs, and *in vitro* cell resistance models, which highlights their broad applicability in clinical settings. A comprehensive overview of these miRNA signatures is provided in **Figure 4**. Collectively, these miRNAs—either individually or in combination—can be detected via tissue or liquid biopsy, serving as promising candidates for non-invasive prediction of targeted therapy responses and monitoring of drug resistance development. **Table 5** summarizes miRNAs associated with therapeutic predictive biomarkers in melanoma.

**Table 5:** Summary of miRNA biomarkers associated with melanoma treatment prediction.

miRNA	Sample	Expression pattern	Biological function	Clinical application direction	References
miR-181a/b	Cells	Downregulated	Targets *TFAM*, inhibiting mitochondrial function	Low expression is associated with drug resistance	(54)
miR-34a	Cells	Downregulated	Inhibits the anti-apoptotic protein BCL2, promoting apoptosis	High expression is associated with increased sensitivity of melanoma cells to cisplatin	(55)
miR-204-5p	Cells	Upregulated	Targets *BCL2* and *SIRT1*, promoting the reversal of dacarbazine-induced senescence	High expression is associated with resistance to dacarbazine	(56)
miR-524-5p	Tissue	Downregulated	Inhibits the *MAPK1* pathway and blocks PI3K/AKT signaling	Predicts response to *BRAF* inhibitor therapy (higher expression indicates better efficacy)	(57)
miR-579-3p	Serum	Downregulated	Associated with disease progression and resistance-related features	Predicts response to *BRAF* inhibitor therapy (higher expression indicates longer PFS)	(58)
miR-4488	Serum	Upregulated	Associated with disease progression and resistance-related features	Predicts response to *BRAF* inhibitor therapy (lower expression indicates longer PFS)	(58)
miR-876-3p	Cells	Downregulated	Targets *MAPK1* to inhibit ERK signaling	Higher expression indicates greater sensitivity to vemurafenib	(59)
miR-152-5p	Cells	Upregulated	Targets *TXNIP*, driving tumor cells toward a slow-cycling, highly invasive phenotype	Associated with acquired resistance to vemurafenib	(60)
miR-204-5p miR-211-5p	Cells	Upregulated	Associated with drug tolerance	Associated with acquired resistance to vemurafenib	(61)
miR-4443 miR-4488	Cells	Upregulated	Targets *NES*, remodeling cytoskeleton and enhancing migratory and invasion capabilities	Associated with resistance to MAPK inhibitor	(62)
miR-126-3p	Cells	Downregulated	Targets *ADAM9* and *VEGFA*, driving proliferation, invasion, and drug resistance	Associated with resistance to dabrafenib	(63, 64)
miR-143-3p miR-145-5p	Cells	Upregulated	Targets *FSCN1*, promoting a transition toward an AXL-high phenotype	Associated with resistance to MAPK inhibitor	(65)
miR-579-3p	Cells	Downregulated	Associated with acquired resistance and accompanied by AXL upregulation	Clinical resistance and recurrence	(66)

Upregulation or downregulation indicates higher or lower miRNA expression in resistant models relative to sensitive models, or in the corresponding control group.

### 4.3 MicroRNA-related therapeutic strategies

In addition to their role as predictive biomarkers, miRNAs are being investigated as innovative therapeutic agents because of their significant gene regulatory capabilities. miRNA-based therapies primarily focus on restoring the function of tumor-suppressing miRNAs (miRNA mimics) or inhibiting the activity of oncogenic miRNAs (antagomiRs), with the success of these approaches largely dependent on the development of efficient targeted delivery systems.

#### 4.3.1 Delivery strategies based on extracellular vesicle

EVs, as natural endogenous nanocarriers, exhibit superior biocompatibility and low immunogenicity, rendering them an optimal platform for miRNA delivery. In a study involving a *BRAF* V600E mutant melanoma model, re-expressed miR-195-5p was packaged into tumor cell–derived EVs and subsequently taken up by neighboring tumor cells, resulting in reduced expression of *CCND1* and yes1 associated transcriptional regulator (*YAP1*). Combined with MAPK inhibitor therapy, this strategy further suppressed *BCL2L1*, increased apoptosis, and markedly delayed tumor growth and recurrence in murine models (67).

#### 4.3.2 Delivery strategies based on synthetic nanoparticles

To address the *in vivo* degradation of miRNAs, researchers have developed various functionalized nanoparticles. Ding et al. developed a DR5–TAT–modified PHRD polymeric micelle system (Co-PHRD) to deliver miR-34a and achieve intracellular release within tumor cells. Through inhibition of sirtuin 1 (*SIRT1*), E2F transcription factor 3 (*E2F3*), and *ZEB1*—key regulators in *TP53*-associated tumor-suppressive processes—this strategy markedly increased apoptosis in melanoma cells (68). Separately, Ma et al. designed redox-responsive quasi-mesoporous magnetic nanospheres (rMMNs) that disassembled in glutathione (GSH)-rich environment of tumor tissues, releasing miR-30a-5p. In an ocular melanoma model, this treatment induced tumor cell apoptosis by inhibiting E2F transcription factor 7 (*E2F7*), promoted polarization of tumor-associated macrophages (TAMs) toward an antitumor M1 phenotype, and triggered a Fenton reaction, thereby producing synergistic effects between gene therapy and immune activation (69).

In melanoma, miRNAs also show potential as adjuncts for overcoming resistance to targeted therapy. One study engineered CSPG4 antibody-targeted chitosan nanoparticles (Ab-CS126) to deliver chemically modified miR-126 mimics (OMe-miR-126) for enhanced stability. In dabrafenib- or vemurafenib-resistant melanoma models, including metastatic settings, this targeted delivery system, in conjunction with a PI3K/AKT inhibitor, effectively suppressed tumor growth by downregulating phosphoinositide-3-kinase regulatory subunit 2 (*PIK3R2*) and significantly reduced liver and lung metastases (63). Another study delivered lipid nanoparticles (LNP-miRs) co-encapsulating miR-204-5p and miR-199b-5p in a *BRAF*-mutant melanoma model. By downregulating *VEGFA*, transforming growth factor beta 1 (*TGFB1*), and C-C motif chemokine ligand 5 (*CCL5*)/C-X-C motif chemokine ligand 2 (*CXCL2*)—key factors in drug resistance escape—and inhibiting the recruitment and polarisation of pro-tumor macrophages, this approach enhanced the efficacy of MAPK inhibitors and delayed the emergence of acquired resistance (70).

#### 4.3.3 Application potential of miRNA replacement therapy

Several studies have focused on replacement therapy using tumor-suppressive miRNA mimics. It has been observed that miR-137 expression is downregulated in advanced melanoma, whereas expression of its target gene T-box transcription factor 3 (*TBX3*) is elevated. *TBX3* transcriptionally represses E-cadherin, a key cell adhesion molecule whose loss promotes EMT and metastasis. Delivery of miR-137 mimics effectively reduced *TBX3* levels, thereby alleviating its transcriptional repression of E-cadherin and restoring cell adhesion. This process significantly inhibited tumor cell migration and invasion, suggesting that miR-137 replacement therapy may hold significant potential for suppressing melanoma metastasis (71). Moreover, a study utilizing a canine model of spontaneous malignant melanoma demonstrated that the intratumoral delivery of a double-stranded miR-634 analog effectively induced apoptosis in tumor cells. Partial remission or disease stabilization was observed in seven clinical cases without significant adverse reactions, indicating promising translational potential (72).

#### 4.3.4 Challenges and opportunities in miRNA therapeutics

Despite these promising preclinical results, several challenges must be addressed before miRNA-based therapies can enter routine clinical use. These include off-target effects, potential immunogenicity of synthetic miRNA mimics or delivery vehicles, inefficient *in vivo* delivery to target tissues, and the complexity of miRNA regulatory networks that may lead to unintended biological consequences. Ongoing research focuses on optimizing delivery systems (e.g., through surface modification of nanoparticles with targeting ligands), improving miRNA stability through chemical modifications, and developing combination strategies that synergize with existing immunotherapies or targeted agents.

In conclusion, despite persistent challenges related to delivery efficiency, targeting specificity, and safety, the studies referenced have preliminarily confirmed the significant potential of miRNA therapy as an innovative treatment strategy for melanoma. This potential is evident whether miRNA therapy is employed as a monotherapy or in conjunction with existing treatments, thereby opening new pathways for overcoming treatment resistance and improving patient prognosis. **Table 6** summarizes upregulated and downregulated miRNAs associated with treatment strategies in melanoma.

**Table 6:** Summary of miRNAs associated with melanoma treatment strategies.

miRNA	Delivery system	Biological function	Clinical application direction	References
miR-126	Anti-CSPG4 antibody–targeted chitosan nanoparticles (Ab-CS126)	Downregulates *PIK3R2*, suppressing tumor growth	In dabrafenib- or vemurafenib-resistant melanoma models, reduces liver and lung metastases	(63)
miR-195-5p	Exosomes	Downregulates *CCND1* and *YAP1*	In combination with MAPK inhibitors, further suppresses *BCL2L1* and enhances tumor cell death	(67)
miR-34a	DR5–TAT–modified PHRD polymeric micelles (Co-PHRD)	Targets *SIRT1*, *E2F3*, and *ZEB1*, modulating *TP53*-associated tumor-suppressive processes	Promotes apoptosis in melanoma cells	(68)
miR-30a-5p	Redox-responsive quasi-mesoporous magnetic nanospheres (rMMNs)	Targets *E2F7* to induce tumor cell apoptosis and promotes M1-like macrophage polarization and triggers a Fenton reaction	Synergistic gene therapy and immune activation in ocular melanoma models	(69)
miR-204-5p	Lipid nanoparticles (LNPs)	Targets *VEGFA*, *TGFB1*, *CCL5*, and *CXCL2*, inhibiting recruitment and polarization of pro-tumor macrophages	In combination with MAPK inhibitors, enhances therapeutic response and delays tumor regrowth	(70)
miR-199b-5p				
miR-137	Mimic	Targets *TBX3*, relieving its transcriptional repression of E-cadherin and restoring cell adhesion	Inhibits melanoma metastasis	(71)
miR-634	Mimic	Downregulates *SLC1A5*, *NFE2L2*, and *BIRC5*, inducing apoptosis	Induces tumor cell apoptosis in a canine spontaneous malignant melanoma model.	(72)

## 5 The immunomodulatory roles of miRNAs in melanoma

From an immunological perspective, increasing evidence has identified miRNAs as critical regulators of the TIME in melanoma. MicroRNAs modulate anti-tumor immune responses through multiple mechanisms. They regulate the expression of immune checkpoint molecules such as programmed death-ligand 1 (PD-L1) and cytotoxic T-lymphocyte-associated protein 4 (CTLA-4) in tumor cells and immune cells. They also influence the recruitment, differentiation, and function of various immune cell populations including T cells, regulatory T cells (Tregs), myeloid-derived suppressor cells (MDSCs), and tumor-associated macrophages. Furthermore, they can be transferred between cells via EVs, enabling intercellular communication that shapes the immune landscape (14, 15). These immunomodulatory functions form the basis for the potential of miRNAs as predictive biomarkers for immunotherapy response, diagnostic indicators of immune status, and prognostic tools that reflect the immune contexture of tumors.

### 5.1 MicroRNAs as immune-related diagnostic biomarkers

Beyond their direct association with tumor cells, specific miRNAs reflect the composition and status of the TIME, offering potential as immune-related diagnostic biomarkers. For instance, miR-155, which is highly expressed in activated immune cells, has been associated with the degree of tumor-infiltrating lymphocyte (TIL) infiltration in melanoma, suggesting its potential as an immune-related biomarker of the tumor microenvironment (73). Similarly, miR-146a-5p, a key regulator of immune responses, is differentially expressed in melanoma tissues with high versus low immune infiltration (74). The concept of liquid biopsy immune monitoring has gained traction, with circulating miRNAs serving as proxies for intratumoral immune activity. Audrito et al. demonstrated that miR-17-5p directly regulates PD-L1 expression in melanoma, with elevated plasma miR-17-5p levels in patients harboring PD-L1-positive metastatic lesions, suggesting its utility as a non-invasive biomarker for PD-L1 status (75). These findings extend the diagnostic utility of miRNAs beyond tumor detection to encompass immune profiling, positioning them as versatile tools for precision oncology.

### 5.2 MicroRNAs associated with immune modulation and prognosis

The TIME is a critical determinant of melanoma prognosis, and miRNAs that regulate immune responses have emerged as powerful prognostic indicators. By modulating immune checkpoint expression, immune cell infiltration, immunosuppressive cell function and immune evasion, these miRNAs influence patient survival outcomes independently of traditional clinicopathological factors.

MicroRNAs directly targeting immune checkpoint molecules have been extensively studied. Bioinformatics tools have both predicted and experimentally validated a set of miRNAs that directly target the 3’ UTR of *CD274*, which encodes PD-L1. Jung et al. demonstrated that miR-181a-3p and miR-223-3p, delivered by IL-2-engineered T-cell-derived EVs, enhance CD8^+^ T cell-mediated killing of melanoma cells by downregulating PD-L1 expression, indicating that measuring their expression in T cells holds potential for predicting patients’ OS (76).

Beyond direct regulation of immune checkpoints, miRNAs also influence the recruitment and function of immune cells within the TIME, correlating with melanoma progression and prognosis. Notably, miRNAs often exhibit “context-dependent functions” in immune regulation. For example, miR-155 exhibits dual immunomodulatory functions in melanoma: it enhances CD8^+^ T cell effector function in a TCR stimulation-dependent manner (73), while also regulating Treg immunosuppressive activity through CTLA-4 silencing (77). Conversely, a cluster of miRNAs including let-7e, miR-125a, miR-99b, miR-146b, and miR-125b—collectively termed “MDSC-miRs”—has been shown to reprogram CD14^+^ monocytes into immunosuppressive MDSCs, with elevated plasma levels correlating with poorer survival outcomes in advanced melanoma patients (78). Certain circulating miRNAs have been linked to immune microenvironment heterogeneity and clinical outcomes: miR-150 and miR-342 are associated with favorable outcomes, while miR-149 and miR-1914 correlate with poor prognosis and impaired immune surveillance (79).

Additionally, recent evidence has linked metastatic progression to immune evasion mechanisms mediated by miRNAs. As discussed previously, exosomal transfer of miR-146a-5p to astrocytes remodels the brain metastatic microenvironment, establishing an inflammatory niche that facilitates tumor growth via *NUMB*/Notch-mediated cytokine release (42). Additionally, miR-146a-5p contributes to immune evasion in the melanoma microenvironment by suppressing signal transducer and activator of transcription 1 (*STAT1*)/IFN-γ-mediated anti-tumor immunity (74). This dual role in promoting metastasis and suppressing anti-tumor immunity underscores the interconnected nature of these processes and highlights the potential of such miRNAs as biomarkers for metastatic risk and immune evasion.

### 5.3 MicroRNAs as predictive biomarkers for immunotherapy

ICIs have emerged as a highly promising treatment option for advanced melanoma, yet only a subset of patients achieve durable clinical benefit. Increasing evidence suggests that the efficacy of ICIs is highly dependent on the immune state of the TIME. In general, an immune-infiltrated TIME, characterized by CD8^+^ T-cell infiltration and high IFN-γ expression, provides an important foundation for favorable responses to ICIs (80, 81). In contrast, a non-T-cell-inflamed TIME is often associated with poor immunotherapy responsiveness. Notably, studies in melanoma further indicate that such a poorly infiltrated TIME is not merely a passive absence of immune cells, but may instead be actively shaped by intrinsic malignant-cell programs and linked to inferior clinical benefit from ICIs (81).

In this context, the importance of miRNAs lies not only in their detectable expression changes, but also in their role as key regulators of the TIME. By influencing antigen presentation, IFN-γ signaling, effector T-cell function, immunosuppressive cell populations, and tumor–stromal–immune interactions, miRNA networks can affect the immune state of the TIME and, consequently, the response to ICIs. Therefore, miRNAs are promising biomarkers of immunotherapy not simply because their expression profiles correlate with clinical outcomes, but because they are mechanistically involved in shaping ICI sensitivity. A deeper understanding of these complex regulatory networks will be essential for improving the predictive and translational value of miRNAs in melanoma immunotherapy.

#### 5.3.1 MicroRNAs predicting positive immunotherapy outcomes

Certain miRNAs are linked to enhanced immunotherapy outcomes by augmenting anti-tumor immune responses. Another study identified a distinct “B-cell EV-miRNA” axis: in patients who respond to anti-PD-1 therapy, EVs derived from CD19^+^ B cells are enriched with miR-99a-5p. This miRNA facilitates antibody production and enhances T-cell-mediated anti-tumor effects by regulating the B cell cycle and DNA repair processes, thereby alleviating the inhibition of antibody class-switch recombination (CSR) (82). Additionally, radiotherapy-related immune genes (*DUSP1*, *CXCL13*, *SLAMF7*, *EVI2B*) are associated with immune infiltration patterns and predict better immunotherapy response. High expression of this four-gene signature is associated with better responses to immunotherapy and longer survival, suggesting that miRNAs targeting these genes also have the potential to predict a favorable prognosis in immunotherapy (83).

#### 5.3.2 MicroRNAs predicting negative immunotherapy outcomes and promoting immune evasion

Conversely, certain miRNAs are associated with immunotherapy resistance by inhibiting immune cell function or creating an inhibitory immune microenvironment. In metastatic melanoma, the tumor secretome has been reported to induce miR-155 upregulation. This miRNA, through an AGO2-mediated post-transcriptional silencing mechanism, reduces CTLA-4 expression in Tregs, thereby enhancing the immunosuppressive function of Tregs. This finding suggests that the miR-155-CTLA-4 axis is a potential mechanism of resistance (77).

Furthermore, tumor cells can directly deliver immunosuppressive miRNAs to immune cells via EVs. Vignard et al. demonstrated that CD8^+^ T cells can internalize melanoma-derived exosomes. The miR-498 and miR-3187-3p contained within these exosomes suppress T-cell cytotoxicity by inhibiting TNF-α production and targeting CD45, thereby attenuating T-cell receptor (TCR) signaling (84). Additionally, researchers have proposed that melanoma EVs deliver a specific set of “MDSC-miRs” (including let-7e, miR-125a, miR-99b, miR-146b, and miR-125b) to CD14^+^ monocytes, reprogramming them into MDSCs with immunosuppressive functions. In advanced patients, elevated baseline plasma levels of this miRNA cluster were significantly correlated with shorter OS and PFS following ICI treatment (78).

Specific miRNA signatures linked to metastatic progression and *BRAF* inhibitor resistance also influence cytokine networks and promote immune evasion. Research shows that the miR-99b, miR-125a and let-7e cluster, hosted by sperm acrosome associated 6 (*SPACA6*), is upregulated in *BRAF* inhibitor-resistant models and tumors from short-responder patients. It activates mTOR/NF-κB pathways, promotes the expression of immunosuppressive cytokines, and serves as a target linking drug resistance and immune evasion (85). Moreover, miRNAs downregulated in metastatic melanoma (e.g., miR-141/miR-200/miR-203/miR-205) affect cytokine pathways and immune microenvironment remodeling. In drug resistance, reduced miR-34a/miR-100/miR-125b correlates with *CCL2* overexpression, while miR-200 family downregulation may promote CD8^+^ T-cell exhaustion and immune evasion (86).

### 5.4 The complexity of immunomodulatory miRNA networks

#### 5.4.1 Context-dependent miRNA networks

The immunomodulatory functions of miRNAs in melanoma are complex and context-dependent. As illustrated by the examples above, the same miRNA can exert opposing effects depending on the cell type in which it is expressed. For instance, miR-155, when expressed in effector T cells, enhances their cytotoxicity and promotes anti-tumor immunity (73). However, when upregulated in Tregs, it enhances their immunosuppressive function by silencing CTLA-4, thereby promoting immune evasion (77). Additionally, when expressed in melanoma-derived exosomes, miR-155-5p targets suppressor of cytokine signaling 1 (*SOCS1*) in fibroblasts, activates Janus kinase 2 (JAK2)/signal transducer and activator of transcription 3 (STAT3) signaling and induces a pro-angiogenic cancer-associated fibroblast phenotype (87). A similar context dependence is observed for miR-146a-5p. In tumor cells, it can remodel the brain metastatic microenvironment by targeting *NUMB* (42), whereas in the immune context it suppresses anti-tumor immunity through inhibition of the *STAT1* signaling pathway (74).

These apparently divergent effects likely arise from several factors. First, the same miRNA may be expressed in different immune or stromal cell populations, each with distinct downstream targets and signaling dependencies. Second, miRNA function is further shaped by the local inflammatory milieu and upstream cues, which can dynamically regulate both miRNA expression and target engagement. Third, non-cell-autonomous regulation, particularly EV-mediated transfer, allows the same miRNA to act in different recipient cells and thereby produce distinct biological outputs. Together, these features make miRNA activity highly context-dependent within the melanoma TIME, posing challenges for both mechanistic interpretation and clinical translation (88, 89).

#### 5.4.2 Melanoma subtype-dependent miRNA networks

Current evidence indicates that different melanoma subtypes exhibit marked differences in their baseline miRNA expression profiles. For example, seven differentially expressed miRNAs have been identified between acral and non-acral melanoma (90). In addition, even within CM, 134 differentially expressed miRNAs have been reported between superficial spreading melanoma and nodular melanoma (91).

The subtype specificity of these miRNA signatures is further compounded by the pronounced heterogeneity of the TIME. Compared with CM, MucM generally displays lower IFN-γ signature levels, suggesting weaker baseline antitumor immune activity and a more immunosuppressive TIME overall (92). Similarly, the TIME of acral melanoma (AM) is also more suppressed than that of CM. Previous studies have shown that the immune microenvironment of AM is characterized by reduced CD8^+^ T cells, natural killer (NK) cells, lymphocytes, and γδ T cells, together with Tregs, M2 macrophages, cancer-associated fibroblasts (CAFs), and exhausted T cells, collectively indicating a more immunosuppressive and tumor-promoting microenvironment (93, 94). A similar pattern has also been observed in metastatic melanoma. Studies suggest that miRNA expression programs change in metastatic lesions, which may contribute to metastatic progression (95). At the same time, compared with baseline subcutaneous lesions, progressive brain metastases show reduced infiltration of CD8^+^ effector T cells, accompanied by increased Tregs and M2-like macrophages, as well as upregulation of immunosuppressive signals such as CXCL1, IL-10, T-cell immunoglobulin and mucin-domain containing-3 (*TIM-3*), and lymphocyte-activation gene 3 (*LAG-3*) (96).

Taken together, differences among melanoma subtypes are reflected both in the subtype specificity of miRNA signatures and in the marked heterogeneity of the TIME in which these miRNAs operate. These two layers jointly determine that the biological interpretation, predictive performance, and overall validity of miRNA biomarkers are highly dependent on context and subtype.

#### 5.4.3 Systemic host response-associated miRNA networks

Importantly, the complexity of immunomodulatory miRNA networks extends beyond local tumor-immune interactions and also reflects systemic host responses. In addition to tumor-derived signals, immune cells and host tissues can actively shape the melanoma microenvironment through EV-mediated transfer of miRNAs. For example, M1 macrophages deliver miR-29c-3p via exosomes to suppress melanoma aggressiveness by targeting *ENPP2*, impairing cell migration and invasion (97). Research has also shown that bone marrow mesenchymal stem cell (BMSC)-derived EVs transfer the long non-coding RNA nuclear paraspeckle assembly transcript 1 (*NEAT1*) into macrophages, where it inhibits miR-374a-5p, derepresses leucine-rich repeat-containing G protein-coupled receptor 4 (*LGR4*), and induces M2 polarization, ultimately promoting melanoma progression and lung metastasis (98). Moreover, systemic host responses may contribute to anti-tumor defense, as liver and immune cells release EVs enriched in tumor-suppressive miRNAs, including miR-34a, miR-192, and miR-194, which target the MDM2 and MDM4 axis (99). These findings suggest that immunomodulatory miRNA networks in melanoma should be understood not only as local regulators within the TIME, but also as components of a broader host-tumor communication system.

**Figure 5:**
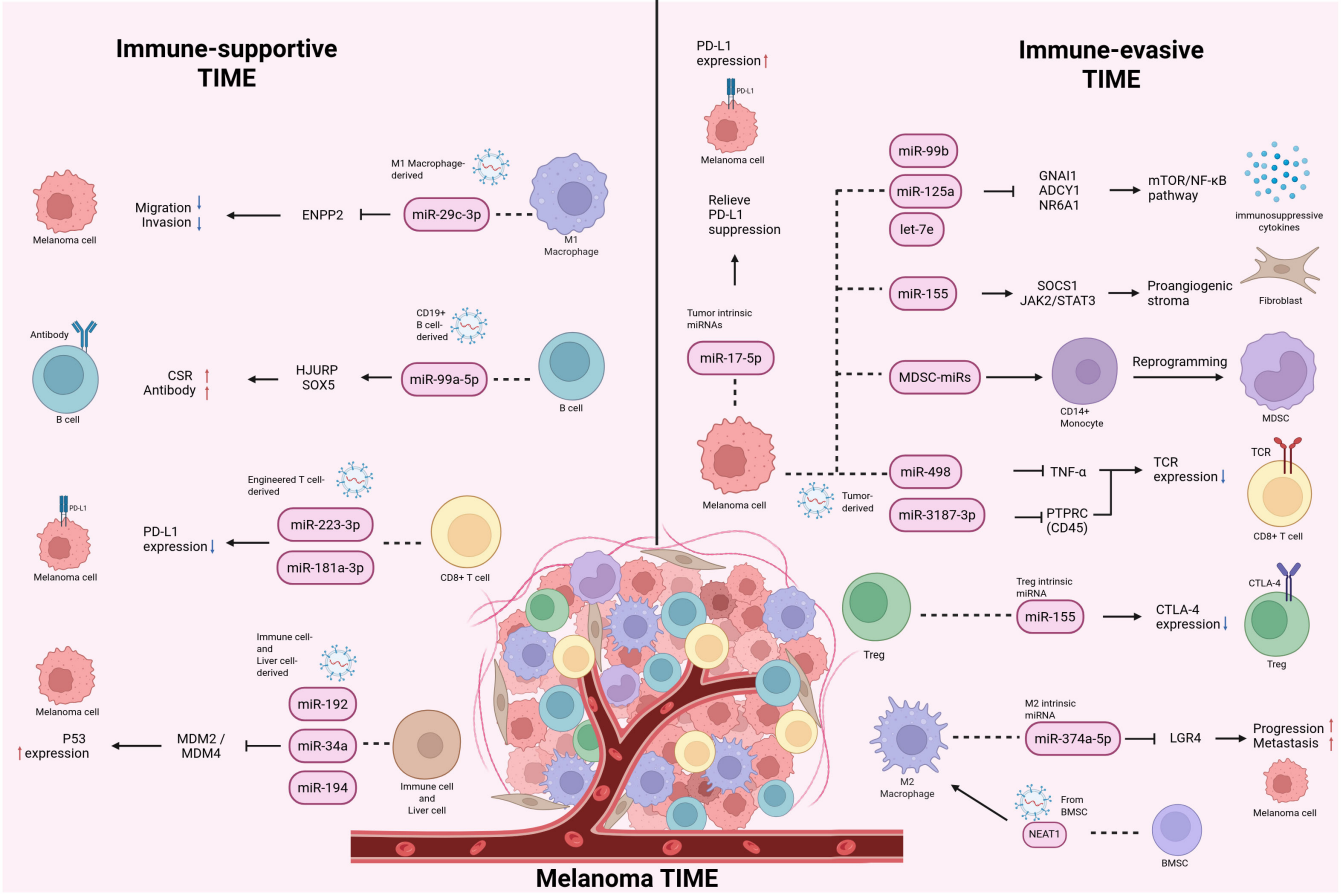
Overview of miRNA-mediated crosstalk within the melanoma tumor immune microenvironment. This figure summarizes representative miRNA-mediated crosstalk within the melanoma TIME. It illustrates how different miRNAs participate in immune-supportive and immune-evasive networks across local and systemic host–tumor communication. Representative downstream regulatory modules include immune checkpoint regulation, stromal activation, and immunosuppressive cytokine programs. Red upward arrows and blue downward arrows indicate promotion and suppression, respectively, of the indicated biological processes. Dashed lines indicate source attribution or intercellular transfer, as appropriate. miRNA, microRNA; TIME, tumor immune microenvironment; PD-L1, programmed death-ligand 1; CTLA-4, cytotoxic T-lymphocyte-associated protein 4; JAK2, Janus kinase 2; STAT3, signal transducer and activator of transcription 3; NF-κB, nuclear factor kappa B; MDSC, myeloid-derived suppressor cell.

In summary, the evidence reviewed in this section highlights the multifaceted immunological significance of miRNAs in melanoma. They not only serve as diagnostic and prognostic markers reflecting the tumor immune state, but also show considerable promise in predicting immunotherapy response. Mechanistically, miRNAs continuously influence the organization and immune state of the TIME by regulating immune cell function, immunosuppressive states, and tumor–host interactions, thereby further affecting patient responses to ICIs. However, these effects should not be interpreted in isolation, but rather within complex regulatory networks. Across different cell types, melanoma subtypes, metastatic sites, and host–tumor communication contexts, miRNAs may exert distinct or even opposing immunological effects. This complexity not only expands their biological significance within the TIME, but also partly explains the context-dependent nature of their clinical utility. **Figure 5** integrates these multilayered miRNA-mediated crosstalk interactions and summarizes representative immune-supportive and immune-evasive regulatory networks within the melanoma TIME. **Table 7** further summarizes immune-related miRNAs associated with melanoma diagnosis, prognosis, and immunotherapy response. Overall, these studies support the view that miRNAs are not only informative biomarkers, but also important regulatory factors involved in shaping the immune landscape of melanoma, and therefore hold substantial research and translational value in precision immunotherapy.

**Table 7:** Summary of immune-related miRNAs in melanoma.

miRNA	Sample	Expression pattern	Biological function	Clinical application direction	References
miR-155	Sorted peripheral blood T-cell subsets	Upregulated	Associated with responsive effector CD8^+^ T cells	Immune microenvironment–associated marker	(73)
miR-146a-5p	Tissue	Upregulated	Targets *STAT1*	Immune microenvironment–associated marker	(74)
miR-17-5p	Plasma	Upregulated	Regulates PD-L1 expression	Immune-status-associated biomarker	(75)
miR-181a-3p	Engineered T cell derived EVs	Downregulated	Downregulates PD-L1 expression, enhancing CD8^+^ T-cell-mediated killing of melanoma cells.	Immunotherapy-related biomarker	(76)
miR-223-3p					
miR-155	Sorted peripheral blood T-cell subsets	Upregulated	Targets CTLA-4	Immunosuppression-related prognostic markers	(77)
MDSC-miRs (including let-7e, miR-125a, miR-99b, miR-146b, miR-125b)	Exosomes	Upregulated	Delivered to CD14^+^ monocytes, reprogramming them into MDSCs with immunosuppressive functions	High expression is associated with poorer prognosis and poorer response to immunotherapy	(78)
miR-99a-5p	Exosomes	Upregulated	Relieves suppression of *HJURP* and *SOX5*, enhancing CSR	High expression is associated with improved response to immunotherapy	(82)
miR-498	Exosomes	Upregulated	Suppresses TNF-α production to attenuate TCR signaling	Potential therapeutic target associated with immune escape	(84)
miR-3187-3p	Exosomes	Upregulated	Targets CD45 to attenuate TCR signaling	Potential therapeutic target associated with immune escape	(84)
miR-99b miR-125a let-7e	Cells	Upregulated	Targets *GNAI1*, *ADCY1*, and *NR6A1* to activate mTOR/NF-κB pathway	High expression is associated with immunosuppressive tumor microenvironment and immune evasion	(85)
miR-200	Tissue	Downregulated	Associated with PD-L1 desensitization	Low expression is associated with CD8^+^ T cell exhaustion and immune evasion	(86)
miR-155	Tissue Exosomes	Upregulated	Targets *SOCS1* to activate the JAK2/STAT3 pathway	Mechanism involved in tumor microenvironment remodeling	(87)
miR-29c-3p	Exosomes	Downregulated	Targets *ENPP2* to inhibit the ENPP2/LPA pathway	M1 macrophages mediated antitumor response	(97)
miR-374a-5p	Cells	Downregulated	Targets *LGR4*	M2 macrophage-driven melanoma progression	(98)
miR-34a miR-192 miR-194	Exosomes	Upregulated	Targets MDM2 and MDM4 axis	Innate immune-mediated antitumor host response	(99)

Upregulated or downregulated indicates higher or lower miRNA expression relative to non-melanoma controls for diagnosis, the favorable-prognosis group for prognosis, and the corresponding reference group in each immunotherapy-related study.

## 6 Challenges and future prospects of miRNA research in melanoma

Although significant progress has been made in miRNA research related to melanoma, multiple critical challenges spanning from basic science to translational medicine remain before comprehensive clinical application can be achieved. These challenges involve technical bottlenecks in sample processing and detection, limitations in understanding the complex regulatory mechanisms of miRNAs, and practical difficulties in developing safe and effective therapeutic strategies.

### 6.1 Current challenges

#### 6.1.1 Lack of standardized protocols for sample processing and detection

The insufficient reproducibility of research findings represents a fundamental obstacle in the current field. Significant variations exist across different studies in sample collection, RNA extraction, and quantitative detection methods, leading to considerable difficulties in cross-study comparison and validation of miRNA expression profiles. This issue is particularly pronounced in the analysis of EV miRNAs. Currently, there is no recognized gold standard for EV isolation: although ultracentrifugation is widely used and yields acceptable quantities, it suffers from limitations such as low specificity, time-consuming procedures, high equipment requirements, and poor inter-laboratory reproducibility. Conversely, while size exclusion chromatography improves purity and integrity, it often results in significant sample dilution, which is unfavorable for downstream applications requiring high concentrations of exosomes (100). The diversity of isolation techniques directly affects the purity and quality of exosomes, thereby interfering with the reliable assessment of miRNA expression levels and hindering their clinical translation as liquid biopsy biomarkers.

#### 6.1.2 Limited efficacy of single miRNAs as biomarkers

Given the high heterogeneity and complex molecular regulatory networks of melanoma, single miRNAs as diagnostic or prognostic markers often lack sufficient sensitivity and specificity. The expression changes of an individual miRNA cannot adequately reflect overall tumor status or prognosis. Therefore, there is an urgent need to establish characteristic expression profiles composed of multiple miRNAs, or to integrate them with molecular data such as protein biomarkers and DNA methylation profiles, to construct multidimensional combined diagnostic and predictive models. This approach aims to enhance detection accuracy, robustness, and their guiding value in personalized treatment.

#### 6.1.3 Credibility of miRNA entities and data quality issues

The reliability of miRNA research is also affected by the quality of source data. Some annotated miRNAs in databases may be based on low-quality sequencing data or even misidentifications, introducing uncertainty into the interpretation of research findings and the subsequent screening of therapeutic targets. For instance, in the authoritative database miRBase release 22.1, only 897 out of 2,349 entries are classified as high-confidence “bona fide human miRNAs” (101). Therefore, before conducting relevant research, it is essential to ensure the reliability of research conclusions by carefully evaluating the authenticity of miRNA entities, validating their expression and function, and relying on high-quality datasets for analysis.

#### 6.1.4 Insufficient depth in understanding miRNA mechanisms of action

Currently, the analysis of miRNA mechanisms in the occurrence and development of melanoma largely remains at the level of phenomenological description, lacking detailed elucidation of their functional mechanisms in specific pathological processes. This limits the comprehensive understanding of complex miRNA regulatory networks and hinders the mechanism-based screening of targets and the rational design of intervention strategies.

#### 6.1.5 Translational bottlenecks in miRNA therapeutic strategies

MicroRNA-based therapeutic strategies have encountered severe challenges in the process of clinical translation, which are primarily concentrated on three core issues: delivery efficiency, off-target effects, and safety.

Firstly, an ideal delivery system must simultaneously meet multiple stringent requirements: protecting miRNA mimics or antisense strands from premature degradation in the bloodstream, achieving enrichment in target tissues, and effectively promoting cellular uptake (102). Secondly, the inherent “multi-target” regulatory characteristic of miRNAs makes off-target effects almost impossible to completely avoid, and therapeutic effects may spread to non-target tissues, causing systemic side effects (101). The most cautionary example is the failure of the miR-34a mimic MRX34 in a phase I clinical trial for melanoma: the trial was prematurely terminated due to severe immune-related adverse events in patients, including four deaths (101, 102). This case underscores the significant risks and challenges faced by miRNA therapies in the transition from the laboratory to the clinic, particularly highlighting issues of off-target effects in non-target tissues, the “last-mile” problem of ensuring endocytosis and functional release in specific target cells, and the difficulty in precisely dosing and timing miRNA mimics to avoid perturbing cellular homeostasis.

### 6.2 Future prospects

To address the aforementioned challenges and advance miRNA research toward clinical application, future studies should focus on the following key directions.

#### 6.2.1 Standardized detection and isolation systems

There is an urgent need to establish and promote standardized protocols for miRNA detection and EV isolation, ensuring data reproducibility and comparability from the source. Concurrently, efforts should be made to develop the next generation of highly sensitive and specific detection platforms, such as microfluidic chip-based electrochemical biosensors or digital PCR technology, aiming to achieve automation and integration of sample preparation and *in-situ* detection, thereby enhancing the clinical accessibility of these technologies.

#### 6.2.2 Advance large-scale clinical validation studies

Well-designed, large-scale, multicenter prospective cohort studies should be actively conducted to systematically evaluate the clinical application value of specific miRNA combinations or signatures in early diagnosis, prognostic stratification, and prediction of treatment response for melanoma. Only after rigorous clinical validation can these molecular markers be expected to be incorporated into international diagnostic and therapeutic guidelines and truly serve clinical decision-making.

#### 6.2.3 Deepen the analysis of miRNA regulatory networks

From a systems biology perspective, it is necessary to conduct in-depth analyses of the complex regulatory networks of miRNAs in melanoma. The application of single-cell sequencing and spatial transcriptomics will be particularly instrumental in unraveling the cell-type-specific roles of miRNAs within the TIME. Spatial transcriptomics, in particular, enables the resolution of miRNA and target gene expression profiles in different cells while preserving the spatial architecture of the tissue (e.g., cells at the invasive front vs. those in the tumor core). This approach will help clarify the expression and functional roles of miRNAs in specific spatial contexts, revealing their relationships with local immune-cell infiltration and activation, and enabling the mapping of immune–miRNA interaction landscapes to uncover spatially organized intercellular communication networks. Recent advancements in artificial intelligence have significantly enhanced the power of spatial transcriptomics by enabling the integration of multi-modal data (e.g., histology and gene expression) and the discovery of complex spatial patterns that correlate with clinical outcomes, thereby facilitating the identification of clinically relevant miRNA signatures within the tumor microenvironment (103–105). Integrating miRNA studies with analyses of other non-coding RNA molecules such as long non-coding RNAs (lncRNAs) and circular RNAs (circRNAs) will help map a more comprehensive gene regulatory landscape.

#### 6.2.4 Integrate miRNA signatures with immunogenomic profiling

Given the central role of the immune microenvironment in melanoma progression and treatment response, future biomarker development should prioritize the integration of miRNA signatures with immunogenomic profiling. This includes combining circulating miRNA panels with established immune biomarkers such as PD-L1 expression, TMB, and TCR repertoire diversity. Preliminary evidence suggests that such multimodal approaches significantly improve the prediction of immunotherapy response compared to individual biomarkers alone. Longitudinal profiling of circulating immune cell-derived exosomal miRNAs may offer real-time insights into the evolving immune response during treatment, facilitating dynamic treatment adaptation.

## 7 Conclusion

MicroRNAs are increasingly recognized for their multifaceted roles in the precision medicine framework for melanoma. As stable and non-invasive biomarkers, they exhibit significant potential for broad clinical applications. Specific miRNA expression profiles derived from blood, tissue, and EVs not only effectively differentiate melanoma from benign pigmented lesions but also reliably predict patient survival outcomes and metastasis risk. Crucially, their immunomodulatory functions position them as unique biomarkers that reflect the state of the TIME, offering predictive value for immunotherapy response that complements existing immunogenomic markers. Furthermore, miRNA expression patterns offer a molecular basis for personalized treatment decisions, demonstrating substantial potential for predicting chemotherapy sensitivity, targeted therapy response, and immunotherapy efficacy. Emerging therapeutic strategies harnessing miRNAs as immunomodulatory agents—alone or in combination with ICIs—hold promise for reshaping the TIME and overcoming treatment resistance.

Despite current research challenges in standardization, elucidation of mechanisms, and therapeutic translation, miRNAs are anticipated to become crucial elements in melanoma clinical management. This progress has been propelled by ongoing advancements in detection technologies, an enhanced understanding of molecular regulatory networks, the integration of single-cell and spatial technologies to deconvolute cell-type-specific functions, and continuous innovation in delivery systems. Meanwhile, AI-assisted analysis of multi-omic and spatial data will further enhance the discovery of clinically relevant miRNA signatures in melanoma. Future endeavors should prioritize the translation of these promising scientific discoveries into reproducible and scalable clinical tools and therapeutic strategies, ultimately improving long-term outcomes for patients with melanoma and contributing significantly to the advancement of precision oncology.
